# Associations of per- and polyfluoroalkyl substances in follicular fluid with polycystic ovarian syndrome in infertile women may be mediated by sex hormones

**DOI:** 10.3389/fpubh.2025.1526918

**Published:** 2025-06-10

**Authors:** Yating Bian, Yingxiao Yue, Yufan Cheng, Dan Wang, Lu He, Peixia Yan, Huan Song, Tong Wang, Weidong Zhou, Xu Zhang, Zhizhen Pan, Liangpo Liu

**Affiliations:** ^1^Department of Public Health Laboratory Sciences, School of Public Health, Shanxi Medical University, Shanxi, China; ^2^Changping Center for Disease Prevention and Control, Changping, Beijing, China; ^3^Reproductive Medicine Center, The First Affiliated Hospital of Xiamen University, School of Medicine, Xiamen University, Xiamen, China; ^4^Fujian Provincial Key Laboratory of Ecology-Toxicological Effects and Control for Emerging Contaminants, Key Laboratory of Ecological Environment and Information Atlas (Putian University) Fujian Provincial University, Putian University, Putian, China; ^5^Xiamen Key Laboratory of Indoor Air and Health, Key Lab of Urban Environment and Health, Institute of Urban Environment, Chinese Academy of Sciences, Xiamen, China; ^6^Key Laboratory of Coal Environmental Pathogenicity and Prevention (Shanxi Medical University), Ministry of Education, Shanxi, China

**Keywords:** per-and polyfluoroalkyl substances, follicular fluid, polycystic ovarian syndrome, perfluoro-n-hexanoic acid, steroid hormones

## Abstract

**Objectives:**

Per- and polyfluoroalkyl substances (PFAS) have been associated with polycystic ovarian syndrome (PCOS), however, the evidence is limited. This study aimed to explore the associations between PFAS in the follicular fluid and PCOS, as well as the mediating role of steroid hormones.

**Methods:**

Forty women with PCOS undergoing treatment for infertility and 56 control participants were included in this study. The levels of 24 PFAS in the follicular fluid and sex hormones in serum were measured. The adjusted odds ratios (ORs) and 95% confidence intervals (CIs) for each PFAS were estimated by multivariable logistic regression. Correlation analysis and multiple linear regression revealed the associations between PFAS and steroid hormones. Bayesian kernel machine regression (BKMR) model was utilized to evaluate the associations between joint and individual PFAS exposure and PCOS. Additionally, *in-vitro* experiment with human ovarian granulosa cell line (KGN cells) was conducted.

**Results:**

The study showed that perfluoro-n-octanoic acid (PFOA) and potassium perfluoro-1-octanesulfonate (PFOS) were the dominant PFAS in the follicular fluid samples, with the median concentration of 4.35 ng/mL and 5.22 ng/mL, respectively. Perfluoro-n-hexanoic acid (PFHxA) were correlated with increased incidences of PCOS (medium vs. low tertile: OR = 1.78, 95% CI: 0.18, 17.19). In the cases, a negative relationship was found between PFHxA and luteinizing hormone (LH; β = −0.44, 95% CI: −8.25, −0.03), while a positive relationship was observed between perfluoro-n-heptanoic acid (PFHpA) and LH (β = 0.504, 95% CI: 0.71, 21.31). PFOA was positively associated with estradiol (E_2_; β = 0.76, 95% CI: 1.52, 19.57). The BKMR model indicated that there might be a joint effect between PFAS mixtures and PCOS, with the posterior inclusion probabilities (PIP) of PFHxA was 0.983. In the cell experiments, PFOA, PFOS, and PFHpA exposure decreased the concentration of E_2_ (*p* < 0.05).

**Conclusion:**

PFHxA in follicular fluids was associated with the elevated odds of PCOS, and steroid hormones may play a role in the etiologic connection.

## 1 Introduction

Per- and polyfluoroalkyl substances (PFAS) represent a substantial group of synthetic chemicals consisting of carbon-fluorine bonds (1). Owing to their surface activity, and thermal and acid resistance, PFAS are widely used in industrial products, including stain repellents, textile coatings, aqueous film-forming firefighting foams, cosmetics, and food packaging (2). At present, PFAS have been detected in environmental samples (3), in wild animals (4) and in humans (5). In the general population, exposure to PFAS mainly occurs through the intake of contaminated food, drinking water, and dust (6). Because of rising health-risk concerns, perfluoro-n-octanoic acid (PFOA), potassium perfluoro-1-octanesulfonate (PFOS), their salts, and related compounds have been listed as persistent organic pollutants (POPs) and banned by the Stockholm Convention. However, the production and use of PFAS and related substances still exist in certain industries in China (7). Considerable evidence from toxicological and epidemiological studies indicates that PFAS exposure is associated with multiple reproductive health problems in women (8–10).

Polycystic ovary syndrome (PCOS), which is characterized by persistent anovulation or sparse ovulation, clinical and biochemical excess androgen level, and bilateral or unilateral polycystic ovarian changes, is a common reproductive endocrine disease in women of reproductive years (11, 12). In 2020, it was estimated that 7.8% of Chinese reproductive-age population were affected by PCOS, leading to significant health-related economic challenges in terms of reproductive, metabolic, and psychological complications (13). In particular, it is one of the most common factors of infertility, accounting for ~80% of anovulatory infertility in females (14). Studies have found that PCOS results from multiple factors such as genes and internal and external environment, and endocrine-disrupting chemicals (EDCs) seem to be the main environmental determinants (15). In the cohort study reported by Vagi et al., a correlation between the increased incidence of PCOS and higher serum levels of PFOA and PFOS was observed (16). Meanwhile, a Chinese case-control study explored the associations between PCOS-related infertility and plasma concentrations of PFAS. After adjusting for confounding factors, the plasma concentration of perfluorododecanoic acid (PFDoA) was related to a significantly increased risk of PCOS-related infertility (17).

*In vitro* fertilization (IVF) technology allowed PCOS patients to obtain oocytes with normal morphology, but problems with poor oocyte development potential and low embryo quality still existed (18). The follicular fluid is an important microenvironment for the growth, development, and maturation of oocytes, and an imbalance of metabolites in fluid can affect the quality of oocytes and the subsequent development of embryos (19, 20). Experimental research in oocytes indicated that PFAS had the potential to modulate follicle hormone activity by altering the hypothalamic-pituitary-ovarian regulation, leading to decreased oocyte quality and further impairing female fertility (21, 22). Chronic exposure to PFOS in mice suppresses ovarian hormones production by selectively reducing histone acetylation of the promoter responsible for ovarian steroidogenic acute regulatory proteins (23). In the patients with premature ovarian insufficiency, the levels of PFOS and potassium perfluoro-1-hexanesulfonate (PFHxS) were negatively associated with estradiol (E_2_) and positively associated with follicle-stimulating hormone (FSH) (9). Heffernan et al. found no positive associations between perfluorinated alkyl acids (PFAAs) and steroid hormone levels in 30 PCOS cases (24). Therefore, PFAS may affect the pathogenesis of PCOS by disrupting endocrine function, but the results of existing studies are inconsistent.

Given the highly invasive nature of obtaining follicular fluid samples, the current researches on PFAS exposure mostly focused on blood or urine samples. Compared with blood or urine samples, PFAS in follicular fluid have a direct and substantial effect on the female reproductive endocrine system. Currently, there is insufficient epidemiological evidence to determine whether a relationship exists between PFAS concentrations in follicular fluid and PCOS (24, 25). This study sought to investigate the potential associations of PFAS in follicular fluid with PCOS and hormonal parameters in women undergoing fertility treatment, so as to enhance our comprehension of the impact of PFAS exposure on female reproductive health.

## 2 Materials and methods

### 2.1 Study population

This case-control study was performed within the Reproductive Medicine Center, the First Affiliated Hospital of Xiamen University following approval by the Ethics Committee of the First Affiliated Hospital of Xiamen University. The PCOS subjects were recruited from December 2018 to May 2021 based on the revised 2003 criteria from the Rotterdam (26). According to the criteria, a diagnosis of PCOS was confirmed when at least 2 out of 3 conditions are satisfied: ovulatory dysfunction, biochemical and/or clinical hyperandrogenism, and polycystic ovarian morphology, while ensuring the exclusion of other specific disorders. Inclusion criteria specified that participants were 20–40 years of old and undergoing IVF. Individuals with endocrine disorders, thyroid conditions, ovarian insufficiency, endometriosis, or other systemic diseases were excluded from participation. All the included women were recieving IVF or intracytoplasmic sperm injection (ICSI) treatment because of male factor infertility, tubal factor, or PCOS. The controls without PCOS, who had regular menstrual cycles and typical ovarian morphology, comprised women experiencing tubal factor infertility as well as those undergoing ICSI treatment for male-related issues. They were enrolled at the same center during the same period. Before collecting samples, all participants were provided with the basic information regarding this study, signed consent forms, and filled out a questionnaire.

### 2.2 Samples collection

The subjects refrained from eating from midnight and provided a fasting blood sample on days 2–4 of the menstrual period prior to beginning their IVF treatment. During the process of ovum retrieval, follicular fluid samples were collected. Then the samples were separated by centrifugation at 4,000 rpm for 10 min, and stored at −80°C within 1 h of collection. The levels of anti-Müllerian hormone (AMH), follicle-stimulating hormone (FSH), luteinizing hormone (LH), estradiol (E_2_), testosterone (T), progesterone (P) and prolactin (PRL) were measured using commercially available test kits (Siemens Healthcare Diagnostics Inc.), as described in previous study (27).

### 2.3 Analysis for PFAS

Twenty-four PFAS in follicular fluid were analyzed: PFOA: perfluoro-n-octanoic acid; PFBA: perfluoro-n-butanoic acid; PFPeA: perfluoro-n-pentanoic acid; PFHxA: perfluoro-n-hexanoic acid; PFHpA: perfluoro-n-heptanoic acid; PFNA: perfluoro-n-nonanoic acid; PFDA: perfluoro-n-decanoic acid; PFUnDA: perfluoro-n-undecanoic acid; PFDoDA: perfluoro-n-dodecanoic acid; PFTrDA: perfluoro-n-tridecanoic acid; PFTeDA: perfluoro-n-tetradecanoic acid; PFOS: perfluoro-1-octanesulfonate; PFBS: potassium perfluoro-1-butanesulfonate; PFPeS: sodium perfluoro-1-pentanesulfonate; PFHxS: potassium perfluoro-1-hexanesulfonate; PFHpS: sodium perfluoro-1-heptanesulfonate; 4:2 FTS: sodium 1H,1H,2H,2H-perfluoro-1-hexanesulfonate; 6:2 FTS: sodium 1H,1H,2H,2H-perfluoro-1-octanesulfonate; 8:2 FTS: sodium 1H,1H,2H,2H-perfluoro1-decanesulfonate; PFOSA: perfluorooctane sulfonamid; PFNS: sodium perfluoro-1-non-anesulfonate; PFDS: sodium perfluoro-1-decanesulfonate; N_MeFOSAA: N-Methylperfluorooctanesulfonamidoacetic acid (Linear and branched) and N_EtFOSAA: N-Ethylperfluorooctanesulfonamidoacetic acid (Linear and branched). PFAS standards were purchased from the AccuStandard (New Haven, CT, USA). ^13^C_8_-PFOA was purchased from Cambridge Isotope Laboratories (Andover, MA, USA). ^13^C_4_-MPFOS was acquired from Wellington Laboratories (Guelph, ON, Canada). Detailed information concerning experimental materials and methods can be referred to our earlier publication (28).

Further details of mobile phase and mass spectrometry parameters can be found in Supplementary Tables S1, S2. The limits of detection (LOD) and quantification (LOQ), relative standard deviation, and matrix-spiked recoveries are provided in Supplementary Tables S3, S4. For PFAS concentrations that fell below the LOD, estimations were calculated by dividing the LOD by the square root of 2.

### 2.4 Cell culture

The human ovarian granulosa tumor cell line (KGN) was cultured with Dulbecco's modified Eagle's/F12 medium (DMEM/F12, Procell Life Science & Technology, Wuhan, China), supplemented with 10% fetal bovine serum (FBS, HyClone, Victoria, Australia) and 1% of penicillin/streptomycin (Gibco, NY, USA). Cultures were incubated in a humidified environment containing 5% CO_2_ at 37°C. Cells was exposed to different concentrations of PFOA, PFOS, and PFHpA, which were purchased from J & K Scientific (Beijing, China). The stock solution was created by dissolving powder in dimethyl sulfoxide (DMSO) and stored at −20°C until needed. It was subsequently diluted with DMEM to achieve the target concentration, resulting in the final DMSO concentration of 0.1%. The DMSO also was used as the control for the experiments. The KGN cells were purchased from the Institute of Cell Biology of the Chinese Academy of Sciences (Shanghai, China).

### 2.5 Cell viability (CCK-8 assay)

The KGN cells were seeded in 96-well plates with medium. A blank group (medium only), control group (0 μM PFOA, PFOS, or PFHpA and cells) and treatment group (0.1 μM, 1 μM, 10 μM, 100 μM, 200 μM, 400 μM, or 600 μM PFOA, PFOS, or PFHpA and cells) were set. The KGN cells were subjected to treatment for 24 h, 48 h, and 72 h, respectively. Control cultures consisted of either medium alone or medium supplemented with 0.1% DMSO. At 24 h, 48 h, and 72 h after PFAS administration, a volume of 10 μL of CCK-8 solution was introduced into each well and the cells were subsequently incubated at 37°C for an additional hour. Blank (medium plus CCK-8 without cells and drugs) and negative control groups (medium plus cells and CCK-8 without drugs) were included in the experimental design. Optical density (OD) was taken at 450 nm by a SpectraMAX M5 microplate reader (Molecular Devices, CA, USA). Each experiment was conducted three times.

### 2.6 Hormone measurement in cell medium (ELISA)

The KGN cells were exposed to different concentrations of PFAS (PFOA: 0.1 μM, 1 μM, 10 μM, 400 μM; PFOS: 0.1 μM, 1 μM, 10 μM, 100 μM; PFHpA: 0.1 μM, 1 μM, 10 μM, 400 μM, respectively) for 48 h. Since the KGN cells cannot secrete FSH and LH (29), AMH, E_2_, T and P hormone levels in culture media were quantified using human AMH ELISA kits, E_2_ ELISA kits, T ELISA kits, and P ELISA kits (Huaying Institute of Biotechnology, Beijing, China) by following the manufacturer's protocol, respectively. All samples were run in three times.

### 2.7 Statistical analysis

Shapiro-Wilk test was used to assess the normality of all data. Median and interquartile range (IQR) were used to represent continuous data and n (%) to represent categorical data. The distinctions between the cases and controls were analyzed by *T*-test and Mann-Whitney U test. A chi-square test was used to examine the differences in the categorical variables. In addition, spearman correlation analysis was employed to assess the correlation of each PFAS concentration (detection rate > 70%) in follicular fluid. To evaluate associations between PFAS levels and PCOS, unconditional logistic regression was used to examine the odds ratio (OR) and 95% confidence interval (95% CI). Given that the relationship between PFAS exposure and PCOS may not follow the monotonic linearity, PFAS concentrations were divided into tertiles. This allowed each PFAS analyte to be represented as a set of indicator variables, with the lowest tertile serving as the referent. The multivariate linear regression model was applied to assess potential associations between PFAS concentrations and hormonal characteristics. Based on existing literature and results of logistic analysis, several confounding factors, including age, body mass index (BMI), and employment status were accounted for in the regression model. In the cell experiment, a two-way analysis of variance (ANOVA) with the Tukey test for multiple comparisons was performed to find significant differences between groups.

Because of the strong relationships between certain PFAS, Bayesian kernel machine regression (BKMR) was utilized to evaluate the associations between individual and combined PFAS exposure and PCOS. This model is crafted to assess the health implications of exposure to multiple environmental contaminants (30). To address skewness and reduce the impact of outliers, the natural logarithm-transformed PFAS (Ln-PFAS) was used for all BKMR analyses. Mediation analyses were conducted to examine the mediating role of sex hormones (Mediator, M) on the relationship between single PFAS concentrations (X) and PCOS (Y). The direct effect (DE) was the effects of single PFAS exposure on PCOS without the mediator. The indirect effect (IE) was the effects of single PFAS exposure on PCOS through the mediator. The total effect (TE) was the combination of DE and IE, and IE/DE was the proportion of mediation which represented the proportion of the explanation by M in the association between X and Y.

The statistical analyses were conducted using SPSS (IBM SPSS Statistics 25, IBM Corp., Armonk, NY, USA) and R software version 4.3.2 (http://www.r-project.org). The BKMR model and mediation analysis were implemented using the R packages “bkmr” and “mediation,” respectively (30, 31). *P-*values of < 0.05 was considered to be statistically significant.

## 3 Results

### 3.1 Participant characteristics

Table 1 summarizes the main demographic and clinical hormonal characteristics of the 40 PCOS cases and 56 controls. Women with PCOS had a markedly higher BMI than the controls (23.30 kg/m^2^ and 21.00 kg/m^2^, respectively, *p* < 0.05). In addition, compared with the controls, the PCOS cases showed higher AMH (7.13 ng/mL vs. 4.77 ng/mL), LH (6.34 mIU/mL vs. 3.94 mIU/mL), and LH/FSH (1.06 vs. 0.59), with the *p* < 0.01. No statistically significant differences were observed between the two groups regarding FSH, E_2_, T, P, and PRL.

**Table 1:** Characteristics of the study population (*n* = 96).

**Characteristic**	**Control (*n* = 56)**	**PCOS (*n* = 40)**	***p*_value*^a^***
	**n (%) or Median (IQR)**	**n (%) or Median (IQR)**	
Age (years)	29.50 (27.00–32.00)	29.00 (27.00–31.00)	0.848
BMI (kg/m^2^)	21.00 (19.63–23.15)	23.30 (20.85–24.20)	0.023^*^
Employment status			0.059
Unemployed	9 (16.1)	13 (32.5)	
Employed	47 (83.9)	27 (67.5)	
AMH (ng/mL)	4.77 (3.05–6.07)	7.13 (5.91–9.95)	0.000^***^
FSH (mIU/mL)	6.91 (5.49–8.21)	6.55 (5.39–8.01)	0.590
LH (mIU/mL)	3.94 (2.90–5.22)	6.34 (4.21–9.76)	0.000^***^
LH/FSH	0.59 (0.43–0.75)	1.06 (0.73–1.44)	0.000^***^
E_2_ (pg/mL)	49.87 (39.31–65.45)	41.05 (30.23–56.11)	0.052
T (ng/mL)	19.03 (0.60–28.04)	26.04 (0.57–55.70)	0.099
P (ng/mL)	0.61 (0.43–0.77)	0.51 (0.40–0.70)	0.269
PRL (ng/mL)	13.54 (11.16–18.17)	13.24 (9.51–21.26)	0.675

PCOS, Polycystic ovary syndrome; IQR, Interquartile Range.

a t-Test or Mann-Whitney U test was used to compare median of continuous variables. Chi-square test was used to compare frequencies of categorical variables.

* p < 0.05;

** p < 0.01;

*** p < 0.001.

The bold values indicate statistical significance.

### 3.2 Profiles of PFAS in follicular fluid

The detection frequencies and concentrations of PFAS in follicular fluid are shown in Table 2. Of the 24 PFAS measured in the 96 samples, 18 compounds were detected in the follicular fluid except for PFDoDA, PFNS, PFDS, PFOSA, N_MeFOSAA, and N_EtFOSAA, which were excluded in the following analysis. The detection frequencies of each compound varied, among which the detection frequencies were more than 50% for 14 PFAS, and 100% for PFOA, PFHpA, PFNA, PFDA, PFUnDA, PFOS, PFPeS, PFHxS, and 4:2 FTS. PFOA and PFOS were the dominant PFAS with median concentrations of 4.35 ng/mL and 5.22 ng/mL, respectively. Compared with the controls, infertile women with PCOS had a significantly higher concentration of PFHpA and 6:2 FTS (*p* < 0.05). The median levels of PFPeA and PFHxA were lower among cases than controls (*p* < 0.001; Table 2). Spearman's correlation analysis revealed that most of the PFAS were highly correlated (*p* < 0.05), with coefficients ranging from −0.347 to 0.864 (Supplementary Table S5). PFOA was significantly positively correlated with PFNA, PFDA, PFUnDA, PFTrDA, PFOS, PFHxS, and 6:2 FTS (*p* < 0.05). PFOS demonstrated a significant positive correlation with PFOA, PFNA, PFDA, PFUnDA, PFTrDA, PFHxS, 6:2 FTS, and 8:2 FTS (*p* < 0.05). In addition to the strong correlations observed for traditional longer-chain PFAS (i.e., PFNA, PFDA, and PFUnDA; coefficients: 0.829 to 0.864), noteworthy correlations were also found in PFAS alternatives (i.e., PFPeS and 4:2 FTS), with a correlation coefficient of 0.646.

**Table 2:** Concentrations of PFAS in follicular fluid in the study population (*n* = 96).

**PFAS**	**Detection frequencies**	**Median (IQR)**			
**(ng/mL)**	**(%)**	**Total (*****n*** = **96)**	**Control (*****n*** = **56)**	**PCOS (*****n*** = **40)**	***p_*** **value** ^a^
PFOA	100	4.35 (3.03–5.95)	4.10 (2.97–5.76)	4.83 (3.29–6.04)	0.383
PFBA	9.38	0.45 (0.28–0.72)	0.50 (0.37–0.72)	0.29 (0.27–0.31)	0.143
PFPeA	85.4	0.12 (0.04–0.26)	0.18 (0.09–0.34)	0.08 (0.04–0.16)	0.000^***^
PFHxA	84.4	0.15 (0.05–0.35)	0.23 (0.11–0.42)	0.05 (0.05–0.21)	0.000^***^
PFHpA	100	0.41 (0.33–0.49)	0.38 (0.31–0.48)	0.44 (0.38–0.53)	0.031^*^
PFNA	100	0.75 (0.54–1.08)	0.74 (0.49–1.00)	0.85 (0.57–1.19)	0.204
PFDA	100	0.60 (0.38–0.90)	0.57 (0.37–0.89)	0.60 (0.43–0.92)	0.412
PFUnDA	100	0.48 (0.35–0.69)	0.48 (0.28–0.69)	0.48 (0.36–0.71)	0.683
PFTrDA	71.9	0.20 (0.05–0.27)	0.19 (0.05–0.27)	0.21 (0.15–0.27)	0.296
PFTeDA	24.0	0.16 (0.15–0.18)	0.16 (0.15–0.20)	0.15 (0.14–0.16)	0.032^*^
PFOS	100	5.22 (3.80–7.71)	4.80 (3.34–7.12)	6.02 (4.20–8.24)	0.059
PFBS	6.25	0.27 (0.12–0.51)	0.28 (0.27–0.64)	0.13 (0.10–0.24)	0.275
PFPeS	100	0.79 (0.65–0.96)	0.73 (0.63–0.94)	0.85 (0.70–0.98)	0.175
PFHxS	100	0.65 (0.50–0.90)	0.64 (0.46–0.86)	0.67 (0.53–1.00)	0.407
PFHpS	19.8	0.44 (0.31–0.57)	0.39 (0.26–0.52)	0.52 (0.42–0.87)	0.136
4:2 FTS	100	0.78 (0.67–0.93)	0.77 (0.57–0.93)	0.83 (0.72–0.94)	0.106
6:2 FTS	79.2	0.16 (0.16–0.32)	0.16 (0.16–0.25)	0.26 (0.16–0.35)	0.005^**^
8:2 FTS	92.7	0.08 (0.05–0.14)	0.08 (0.05–0.13)	0.07 (0.05–0.17)	0.984
Σ_18_PFASs		16.06 (11.46–22.93)	15.38 (10.93–22.16)	17.33 (12.56–23.29)	

IQR, Interquartile Range.

a Tests of differencies between controls and cases using Mann-Whitney U test.

* p < 0.05;

** p < 0.01;

*** p < 0.001.

The bold values indicate statistical significance.

### 3.3 Associations between PFAS in follicular fluid and PCOS

Table 3 summarizes the associations between PFAS levels and PCOS. In the adjusted model, middle levels of PFHxA in follicular fluid showed a significant correlation with an increased risk of PCOS (medium vs. low tertile: OR = 1.78, 95% CI: 0.18, 17.19, *p* = 0.013, P for trend = 0.003). Moreover, middle levels of 6:2 FTS were significantly correlated with PCOS (medium vs. low tertile: OR = 0.05, 95% CI: 0.00, 0.86, *p* = 0.039, P for trend = 0.002).

**Table 3:** Odds ratios and 95% confidence interval for PFAS concentrations in follicular fluid associated with PCOS (*n* = 96).

**PFAS**	**Tertiles**	**Crude OR**	***p_*value**	**Adjusted OR*^a^***	***p_*value**
	**(ng/mL)**	**(95%CI)**		**(95%CI)**	
**PFOA**	1st (1.66–3.49)	1.00 (reference)		1.00 (reference)	
	2nd (>3.49–5.15)	1.32 (0.06, 30.71)	0.863	1.14 (0.03, 42.82)	0.944
	3rd (>5.15–30.99)	0.25 (0.02, 3.02)	0.276	0.21 (0.02, 2.89)	0.242
	*P* for trend**^b^**	0.711		0.679	
**PFPeA**	1st (0.04–0.07)	1.00 (reference)		1.00 (reference)	
	2nd (>0.07–0.19)	11.87 (1.37, 103.08)	0.025^*^	1.84 (0.13, 25.26)	0.029^*^
	3rd (>0.19–0.63)	2.35 (0.31, 17.62)	0.405	3.24 (0.33, 32.34)	0.316
	*P* for trend	0.074		0.455	
**PFHxA**	1st (0.05–0.08)	1.00 (reference)		1.00 (reference)	
	2nd (>0.08–0.26)	13.26 (1.81, 97.05)	0.011^*^	1.78 (0.18, 17.19)	0.013^*^
	3rd (>0.26–2.39)	0.62 (0.08, 4.97)	0.651	0.72 (0.06, 8.90)	0.798
	*P* for trend	0.001^**^		0.003^**^	
**PFHpA**	1st (0.20–0.36)	1.00 (reference)		1.00 (reference)	
	2nd (>0.36–0.46)	0.12 (0.01, 1.23)	0.074	0.10 (0.01, 1.46)	0.093
	3rd (>0.46–2.21)	0.96 (0.15, 6.17)	0.968	1.26 (0.16, 9.93)	0.826
	*P* for trend	0.063		0.161	
**PFNA**	1st (0.21–0.62)	1.00 (reference)		1.00 (reference)	
	2nd (>0.62–0.94)	0.15 (0.00, 9.76)	0.374	0.20 (0.00, 24.96)	0.514
	3rd (>0.94–10.64)	0.03 (0.00, 0.56)	0.020^*^	0.03 (0.00, 0.63)	0.025^*^
	*P* for trend	0.114		0.185	
**PFDA**	1st (0.13–0.44)	1.00 (reference)		1.00 (reference)	
	2nd (>0.44–0.79)	1.09 (0.02, 77.59)	0.970	1.04 (0.01, 107.88)	0.988
	3rd (>0.79–42.46)	1.73 (0.10, 29.05)	0.704	2.04 (0.08, 55.70)	0.672
	*P* for trend	0.389		0.276	
**PFUnDA**	1st (0.11–0.37)	1.00 (reference)		1.00 (reference)	
	2nd (>0.37–0.62)	0.98 (0.01, 97.34)	0.992	0.91 (0.01, 104.22)	0.970
	3rd (>0.62–7.93)	6.72 (0.28, 160.72)	0.240	5.96 (0.15, 234.01)	0.340
	*P* for trend	0.570		0.808	
**PFTrDA**	1st (0.05–0.12)	1.00 (reference)		1.00 (reference)	
	2nd (>0.12–0.25)	1.01 (0.04, 23.71)	0.994	2.43 (0.04, 134.08)	0.666
	3rd (>0.25–1.06)	1.95 (0.12, 32.40)	0.642	2.52 (0.07, 90.03)	0.614
	*P* for trend	0.184		0.372	
**PFOS**	1st (1.55–4.17)	1.00 (reference)		1.00 (reference)	
	2nd (>4.17–6.57)	0.51 (0.05, 5.83)	0.588	0.56 (0.04, 8.06)	0.672
	3rd (>6.57–24.62)	1.62 (0.16, 15.94)	0.681	2.19 (0.17, 28.63)	0.549
	*P* for trend	0.953		0.948	
**PFPeS**	1st (0.39–0.68)	1.00 (reference)		1.00 (reference)	
	2nd (>0.68–0.91)	0.05 (0.00, 0.67)	0.024^*^	0.03 (0.00, 0.75)	0.033^*^
	3rd (>0.91–1.36)	0.13 (0.01, 1.54)	0.106	0.09 (0.01, 1.63)	0.104
	*P* for trend	0.114		0.091	
**PFHxS**	1st (0.21–0.56)	1.00 (reference)		1.00 (reference)	
	2nd (>0.56–0.78)	5.60 (0.23, 134.378)	0.288	3.74 (0.07, 211.91)	0.523
	3rd (>0.78–2.34)	2.69 (0.22, 32.39)	0.435	3.34 (0.20, 56.70)	0.403
	*P* for trend	0.211		0.280	
**4:2 FTS**	1st (0.41–0.72)	1.00 (reference)		1.00 (reference)	
	2nd (>0.72–0.87)	0.75 (0.07, 8.49)	0.816	1.77 (0.09, 36.56)	0.712
	3rd (>0.87–1.29)	3.29 (0.30, 35.72)	0.327	4.66 (0.23, 93.66)	0.315
	*P* for trend	0.328		0.565	
**6:2 FTS**	1st (0.16–0.17)	1.00 (reference)		1.00 (reference)	
	2nd (>0.17–0.25)	0.15 (0.02, 1.11)	0.063	0.05 (0.00, 0.86)	0.039^*^
	3rd (>0.25–0.85)	2.77 (0.18, 43.62)	0.470	2.17 (0.08, 59.96)	0.647
	*P* for trend	0.002^**^		0.002^**^	
**8:2 FTS**	1st (0.05–0.06)	1.00 (reference)		1.00 (reference)	
	2nd (>0.06–0.11)	1.27 (0.18, 8.82)	0.808	0.89 (0.11, 7.57)	0.917
	3rd (>0.11–0.36)	0.40 (0.04, 4.32)	0.454	0.25 (0.02, 3.36)	0.298
	*P* for trend	0.749		0.486	

OR, odds ratio; CI, confidence interval.

a Adjusted for age, BMI and employment status.

b P value for test of trend across quartiles.

* p < 0.05;

** p < 0.01;

*** p < 0.001.

The bold values indicate statistical significance.

### 3.4 Associations between PFAS in follicular fluid and steroid hormones

Supplementary Table S6 presents the findings of the correlation analysis, which revealed no significant correlation between the PFAS in follicular fluid and sex hormones. After adjusting for confounding factors in multivariate linear regression, detailed beta coefficients (β) and 95% CI are listed in Table 4. In the cases, PFOA levels in follicular fluid were positively associated with serum E_2_, with ~0.76 pg/mL increase per unit increase of PFOA (95% CI: 1.52, 19.57). A significant positive association between serum PFHpA and LH was observed (β = 0.50, 95% CI: 0.71, 21.31), while PFHxA was in the opposite (β = −0.44, 95% CI: −8.25, −0.03). Among the controls, PFOS was positively associated with P (β = 0.62, 95% CI: 0.02, 0.42), but negatively associated with the AMH (β = −0.59, 95% CI: −1.11, −0.09).

**Table 4:** Adjusted linear associations (β, 95% CI) of PFAS and steroid hormones (*n* = 96).

**PFAS**	**Steroid Hormones**, β **(95% CI)**							
	**AMH (ng/mL)**		**FSH (mIU/mL)**		**LH (mIU/mL)**		**LH/FSH**	
**(ng/mL)**	**Control**	**PCOS**	**Control**	**PCOS**	**Control**	**PCOS**	**Control**	**PCOS**
PFOA	−0.11 (−0.48, 0.32)	0.15 (−1.03, 1.68)	−0.41 (−0.52, 0.06)	−0.44 (−0.81, 0.16)	−0.10 (−1.71, 1.25)	−0.54 (−1.97, 0.14)	−0.07 (−0.27, 0.21)	0.58 (−0.08, 1.21)
PFPeA	−0.13 (−10.11, 4.38)	0.11 (−21.77, 34.44)	0.09 (−3.75, 6.79)	0.05 (−9.10, 10.98)	0.04 (−23.87, 29.54)	0.33 (−6.03, 37.85)	0.05 (−3.75, 4.93)	−0.10 (−15.97, 10.69)
PFHxA	0.04 (−1.98, 2.55)	−0.11 (−6.58, 3.94)	−0.31^*^(−3.34, **−0.05)**	−0.22 (−2.79, 0.98)	−0.12 (−11.06, 5.61)	−0.44^*^(−8.25, **−0.03)**	−0.10 (−1.76, 0.95)	0.08 (−2.07, 2.92)
PFHpA	0.03 (−2.65, 3.27)	0.27 (−5.91, 20.47)	0.01 (−2.11, 2.20)	0.35 (−1.36, 8.07)	−0.21 (−17.68, 4.14)	0.50^*^ **(0.71, 21.31)**	−0.20 (−2.84, 0.70)	−0.18 (−8.59, 3.92)
PFNA	−0.94 (−8.07, 3.15)	−0.03 (−13.41, 12.71)	1.76 (−0.59, 7.57)	0.36 (−3.12, 6.22)	0.04 (−20.31, 21.03)	0.63 (−4.10, 16.29)	−0.14 (−3.55, 3.16)	−0.62 (−9.69, 2.70)
PFDA	−0.97 (−1.98, 0.73)	0.63 (−1.53, 14.63)	−0.44 (−1.2, 0.77)	0.43 (−1.33, 4.44)	0.23 (−4.53, 5.46)	0.42 (−2.82, 9.80)	0.38 (−0.68, 0.94)	0.12 (−3.28, 4.39)
PFUnDA	2.06 (−0.25, 14.65)	−0.45 (−26.47, 9.98)	−1.56 (−9.55, 1.30)	−0.46 (−9.49, 3.54)	−0.06 (−28.14, 26.78)	−0.57 (−22.49, 5.96)	−0.02 (−4.50, 4.42)	0.13 (−7.52, 9.77)
PFTrDA	0.08 (−11.88, 15.25)	−0.12 (−29.69, 18.06)	0.48 (−2.30, 17.44)	0.34 (−2.75, 14.32)	−0.17 (−61.39, 38.58)	−0.14 (−23.96, 13.31)	−0.15 (−9.71, 6.53)	0.23 (−6.08, 16.57)
PFOS	−0.59^*^(−1.11, **−0.09)**	−0.05 (−0.79, 0.69)	−0.22 (−0.54, 0.20)	−0.15 (−0.31, 0.21)	0.46 (−0.39, 3.36)	−0.35 (−0.85, 0.30)	0.48 (−0.05, 0.56)	0.20 (−0.26, 0.44)
PFPeS	−0.20 (−9.13, 3.01)	0.27 (−7.02, 20.94)	0.06 (−3.77, 5.06)	−0.37 (−8.34, 1.65)	0.05 (−19.79, 24.93)	−0.27 (−16.39, 5.44)	0.02 (−3.44, 3.82)	0.13 (−5.07, 8.2)
PFHxS	0.07 (−3.37, 4.78)	0.02 (−7.99, 8.52)	0.27 (−0.93, 5.00)	−0.14 (−3.53, 2.37)	−0.31 (−24.81, 5.23)	−0.25 (−8.85, 4.05)	−0.33 (−4.15, 0.73)	−0.25 (−5.35, 2.48)
4:2 FTS	0.07 (−5.32, 7.38)	−0.17 (−20.29, 10.21)	−0.25 (−7.57, 1.68)	0.50 (−0.18, 10.72)	−0.21 (−34.00, 12.82)	0.12 (−9.06, 14.75)	−0.16 (−5.12, 2.49)	0.32 (−2.83, 11.64)
6:2 FTS	0.23 (−5.19, 19.64)	−0.20 (−25.92, 13.75)	−0.14 (−12.36, 5.70)	0.25 (−4.49, 9.69)	−0.19 (−64.48, 27.00)	0.5 (−3.46, 27.51)	−0.16 (−10.00, 4.86)	−0.12 (−11.08, 7.74)
8:2 FTS	−0.07 (−15.37, 9.57)	−0.45 (−60.03, 1.24)	0.20 (−2.36, 15.79)	0.10 (−8.74, 13.15)	0.10 (−31.67, 60.24)	−0.25 (−36.70, 11.13)	0.09 (−5.48, 9.45)	−0.05 (−15.92, 13.14)
**PFAS**	**Steroid Hormones**, β **(95% CI)**							
	**E**_2_ **(pg/mL)**		**T (ng/mL)**		**P (ng/mL)**		**PRL (ng/mL)**	
**(ng/mL)**	**Control**	**PCOS**	**Control**	**PCOS**	**Control**	**PCOS**	**Control**	**PCOS**
PFOA	−0.07 (−5.75, 4.70)	0.76^*^ **(1.52, 19.57)**	0.06 (−2.05, 2.47)	0.20 (−4.52, 9.63)	−0.07 (−0.18, 0.14)	0.47 (−0.05, 0.23)	−0.17 (−1.50, 0.82)	0.30 (−1.08, 3.16)
PFPeA	−0.04 (−102.55, 85.88)	0.22 (−101.76, 272.61)	−0.28 (−71.31, 10.19)	0.31 (−38.89, 254.53)	0.16 (−1.62, 4.13)	−0.09 (−3.44, 2.5)	−0.01 (−21.65, 20.11)	0.07 (−37.23, 50.73)
PFHxA	−0.10 (−37.39, 21.40)	0.19 (−20.07, 50.06)	−0.03 (−13.79, 11.63)	0.29 (−7.72, 47.24)	−0.16 (−1.31, 0.49)	−0.03 (−0.59, 0.53)	−0.06 (−7.70, 5.33)	−0.01 (−8.45, 8.03)
PFHpA	−0.16 (−55.64, 21.32)	−0.07 (−100.23, 75.49)	−0.07 (−20.40, 12.88)	0.08 (−55.87, 81.84)	−0.24 (−2.02, 0.33)	−0.14 (−1.74, 1.05)	−0.17 (−13.15, 3.91)	0.10 (−16.20, 25.08)
PFNA	1.01 (−44.51, 101.29)	−0.91 (−160.21, 13.74)	−0.27 (−35.01, 28.04)	−0.47 (−102.09, 34.29)	0.49 (−1.78, 2.67)	−0.23 (−1.63, 1.13)	−0.25 (−17.92, 14.41)	−0.43 (−29.02, 11.86)
PFDA	0.44 (−14.63, 20.64)	0.26 (−36.00, 71.64)	−0.08 (−7.88, 7.38)	−0.30 (−60.28, 24.08)	0.51 (−0.43, 0.65)	0.09 (−0.77, 0.94)	0.39 (−3.25, 4.57)	0.57 (−2.96, 22.33)
PFUnDA	−1.69 (−160.13, 33.59)	0.45 (−66.89, 175.85)	0.37 (−35.61, 48.17)	0.58 (−32.02, 158.24)	−0.74 (−3.86, 2.05)	−0.04 (−1.98, 1.87)	−0.61 (−27.12, 15.83)	−0.04 (−29.85, 27.18)
PFTrDA	0.33 (−102.54, 250.10)	−0.13 (−200.22, 117.79)	−0.27 (−104.03, 48.48)	−0.43^*^(−250.06, **−0.82)**	−0.25 (−7.23, 3.54)	0.06 (−2.26, 2.78)	0.65 (−2.71, 75.46)	−0.42 (−70.95, 3.77)
PFOS	0.17 (−4.73, 8.48)	0.22 (−3.46, 6.33)	−0.46 (−5.14, 0.58)	0.53 (−0.72, 6.96)	0.62^*^ **(0.02, 0.42)**	0.20 (−0.06, 0.10)	−0.53 (−2.90, 0.03)	−0.33 (−1.69, 0.61)
PFPeS	−0.01 (−80.17, 77.59)	0.3 (−42.62, 143.65)	−0.14 (−44.23, 24.00)	0.23 (−38.09, 107.90)	0.14 (−1.66, 3.16)	0.21 (−0.99, 1.96)	−0.15 (−23.65, 11.33)	0.06 (−19.30, 24.46)
PFHxS	−0.06 (−59.41, 46.57)	−0.20 (−70.94, 39.05)	0.01 (−22.59, 23.24)	−0.19 (−56.51, 29.70)	−0.32 (−2.70, 0.53)	−0.31 (−1.22, 0.53)	0.10 (−9.06, 14.43)	0.13 (−10.28, 15.56)
4:2 FTS	−0.06 (−92.26, 72.93)	0.00 (−101.33, 101.81)	−0.03 (−37.92, 33.52)	−0.25 (−122.96, 36.25)	−0.18 (−3.51, 1.54)	−0.01 (−1.64, 1.58)	−0.13 (−23.88, 12.74)	−0.19 (−33.27, 14.45)
6:2 FTS	−0.23 (−237.82, 84.90)	0.01 (−130.15, 134.11)	0.14 (−48.38, 91.18)	0.26 (−57.81, 149.31)	−0.24 (−7.59, 2.26)	−0.11 (−2.41, 1.78)	0.41 (−1.51, 70.03)	0.50 (−6.44, 55.65)
8:2 FTS	0.02 (−152.69, 171.51)	−0.38 (−367.16, 40.90)	−0.05 (−81.46, 58.75)	−0.14 (−212.08, 107.73)	0.08 (−3.64, 6.25)	−0.09 (−3.78, 2.69)	−0.22 (−62.38, 9.48)	−0.39 (−89.34, 6.53)

β, beta coefficients; CI, confidence interval.

* p < 0.05;

** p < 0.01;

*** p < 0.001.

The bold values indicate statistical significance.

### 3.5 BKMR-based models of the association of joint and individual PFAS exposure with PCOS

**Figure 1:**
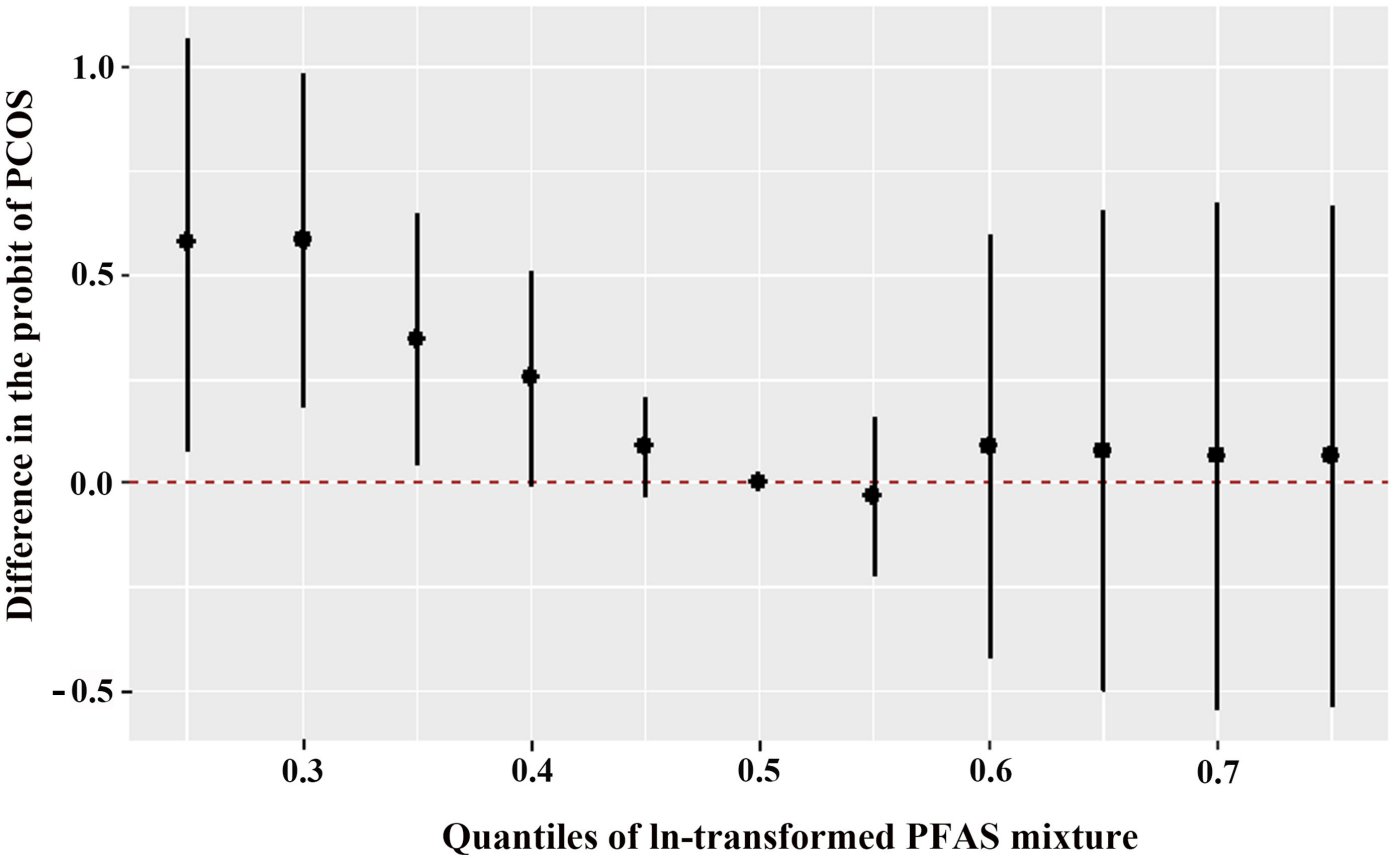
Overall joint associations of PFAS mixture (estimates and 95% confidence intervals) with the PCOS prevalence in Chinese women diagnosed with infertility (*n* = 96). All estimates were adjusted for age, BMI, and employment status. This figure plots the estimated difference in the probit of PCOS when exposures are at a particular percentile (x-axis) in comparison with when exposures are all at the 50th percentile.

**Figure 2:**
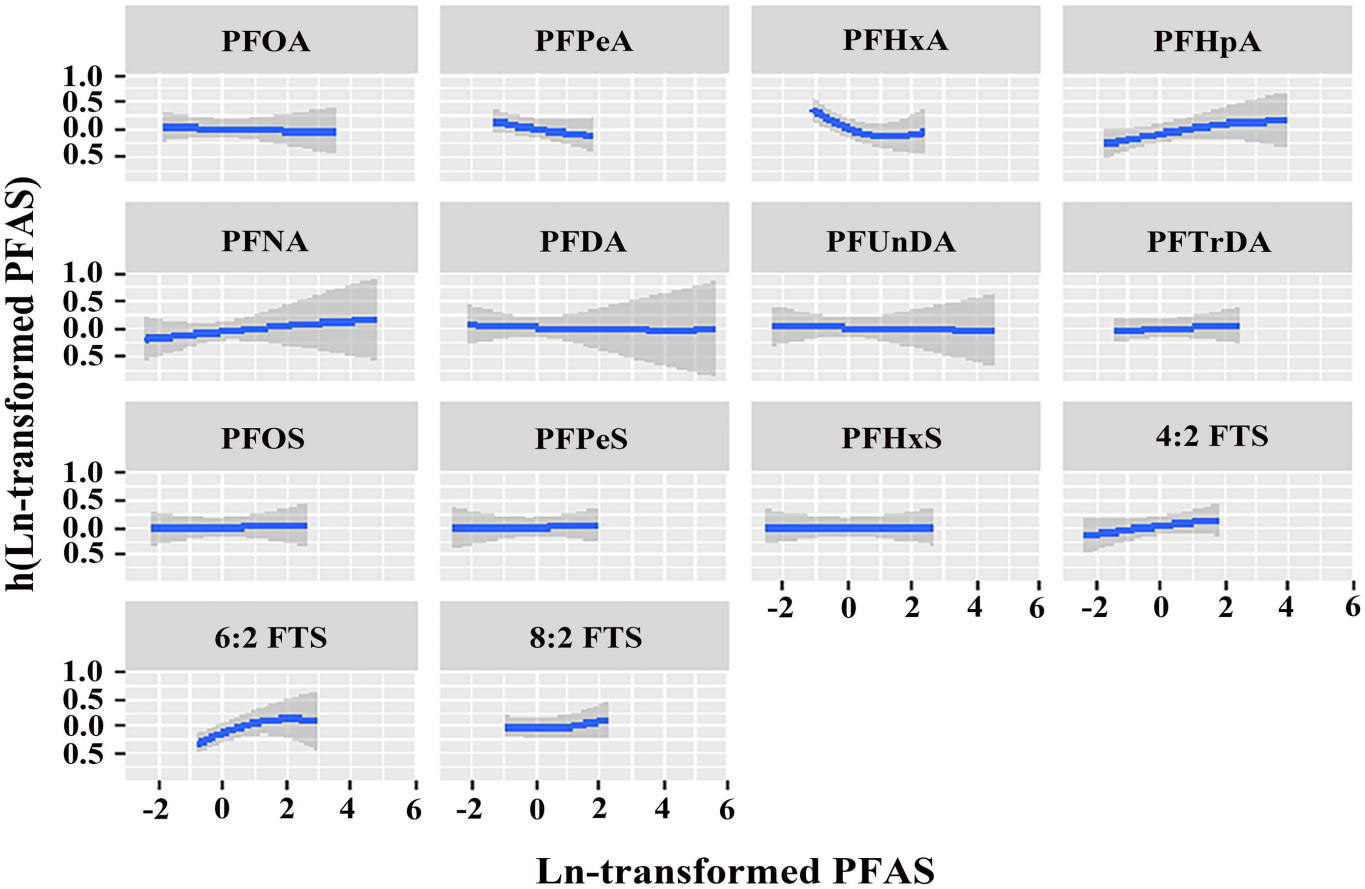
Univariate exposure-response relationship of individual PFAS concentrations (estimates and 95% CIs) with the prevalence of PCOS, with the other pollutants fixed at the median (*n* = 96). All estimates were adjusted for age, BMI, and employment status. The boundaries of the gray areas represent the 95% CIs of the exposure-response relationship.

Figure 1 shows the overall relation between the PFAS mixture and PCOS. The PFAS mixture was negatively associated with the risk of PCOS when PFAS levels were below the 50th percentile. To disentangle which PFAS dominates the overall effect of the mixtures, the posterior inclusion probabilities (PIP) for all PFAS were calculated. PFHxA (PIP = 0.983), 6:2 FTS (PIP = 0.975) and PFPeA (PIP = 0.973) were the main contributors to this trend (Supplementary Table S7). By setting the concentrations of other PFAS to the 50th percentile, the univariate exposure-response functions were displayed. As shown in Figure 2, PFHxA and 6:2 FTS presented a non-linear relationship with PCOS. The associations of the other PFAS with PCOS remained mostly unchanged when holding the compounds at their median.

### 3.6 The mediation effect of LH in the relationship between PFHxA and PCOS

**Figure 3:**
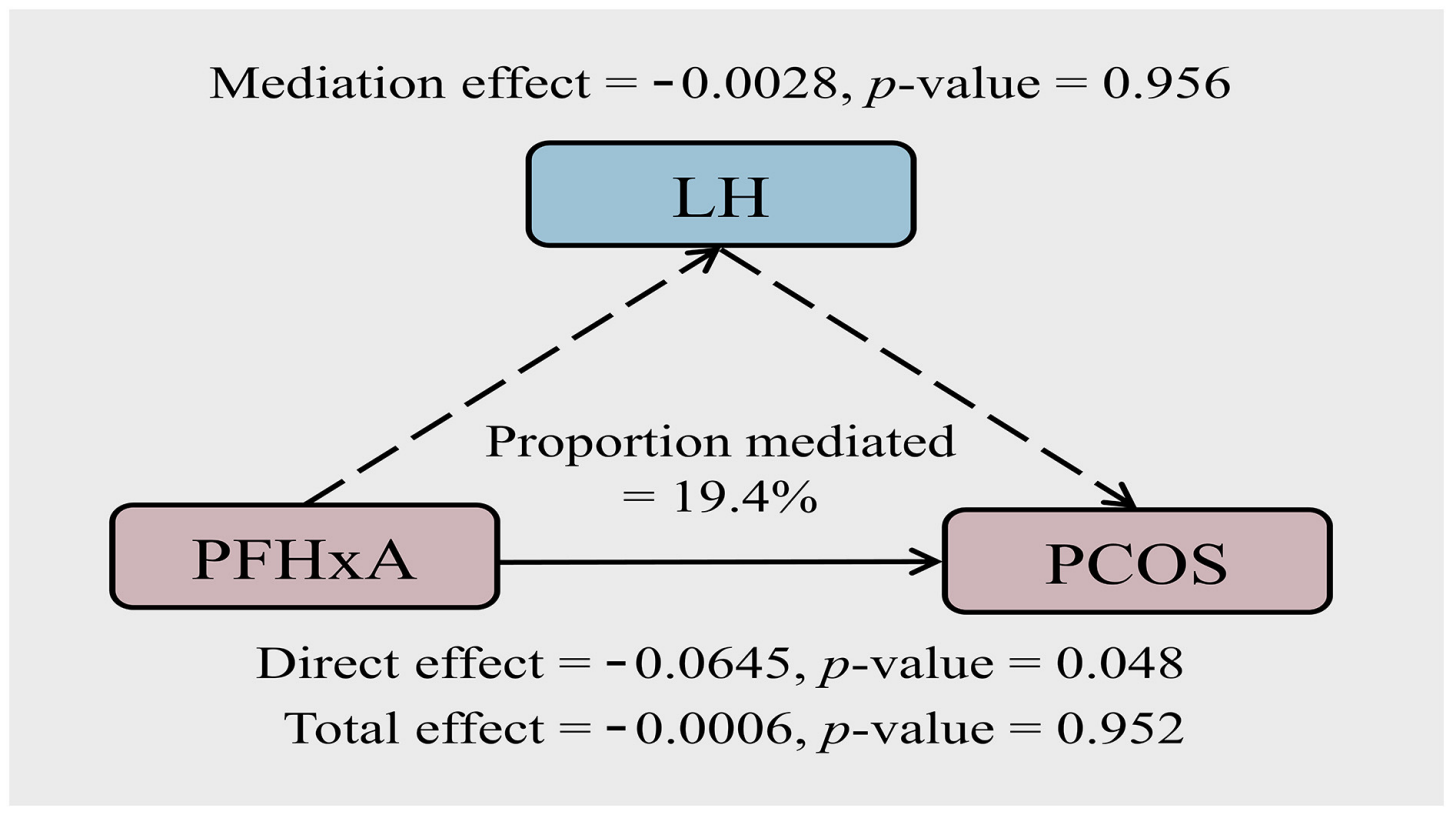
Path diagram of the mediation analysis of LH in the relationship between PFHxA and PCOS. The model was adjusted for age, BMI, and employment status.

Since PFHxA was associated with both LH and PCOS, the mediating role of LH in the association of PFHxA exposure and PCOS was further analyzed. The results are presented in Figure 3. LH explained 19.40% of the association between PFHxA and PCOS. The direct effect of PFHxA was significant (*p* = 0.048); however, the mediation effect of LH was not statistically significant (*p* = 0.956).

### 3.7 The effect of PFOA, PFOS, and PFHpA exposure on KGN cell viability and hormones

**Figure 4:**
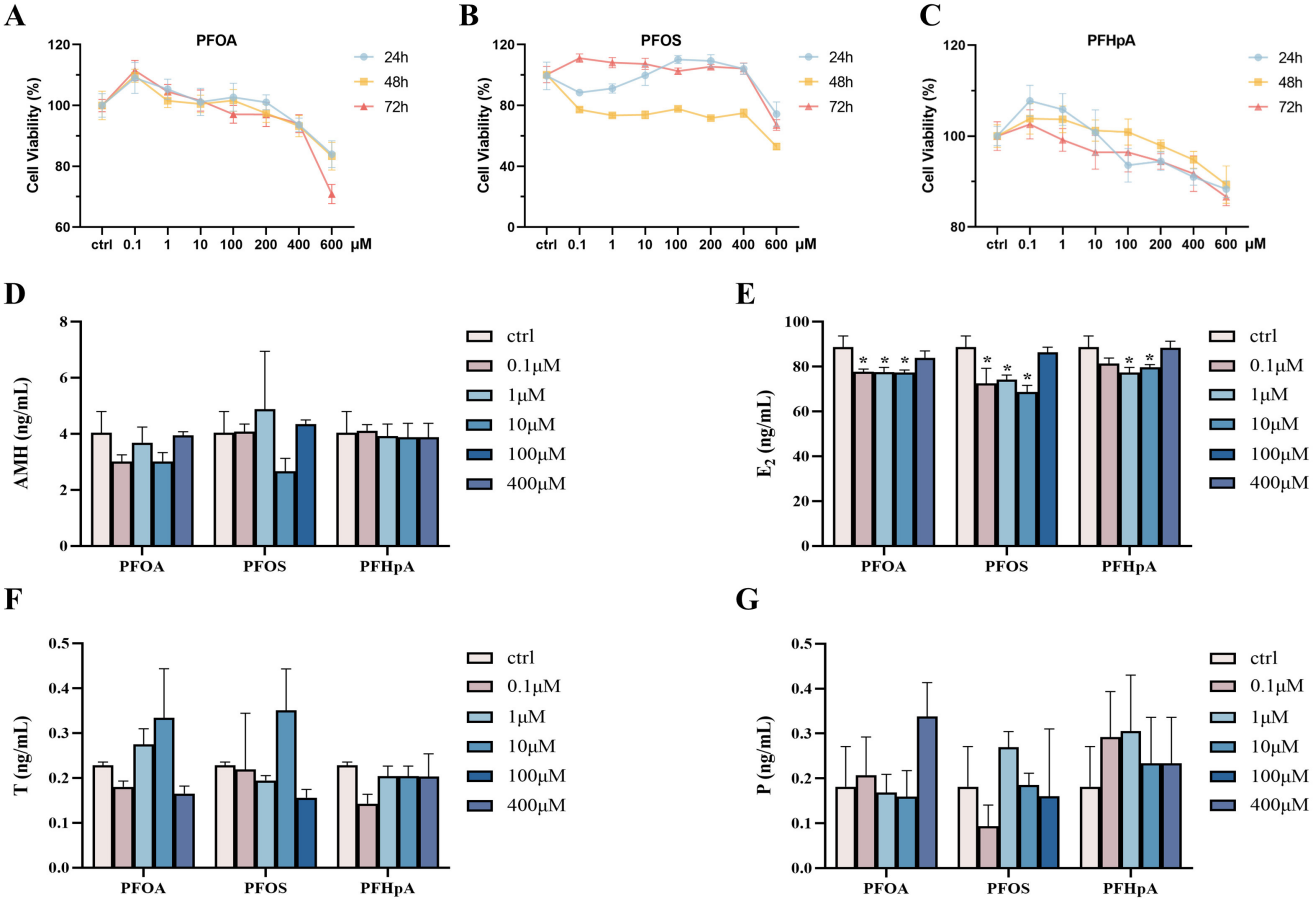
Cytotoxicity induced by PFAS in KGN cell line and the effects of expression of hormone levels. Cell viability was measured in KGN cells after being treated with different concentrations of PFOA **(A)**, PFOS **(B)**, and PFHpA **(C)** for 24 h, 48 h, and 72 h. Bar graphs show levels of AMH **(D)**, E_2_ **(E)**, T **(F)**, and P **(G)** in the cell supernatant, respectively. *p < 0.05; **p < 0.01; ***p < 0.001.

Table 4 indicated a noteworthy association between PFOA, PFOS, PFHpA, and steroid hormones. Consequently, *in vivo* experiments were further employed to determine the effect of PFOA, PFOS, and PFHpA exposure on the KGN cell viability and hormones. The results of the cell viability are shown in Figures 4A–C, and detailed P-values for statistical analyses are listed in Supplementary Table S8. There was a slight increase in cell viability when PFOA and PFHpA were exposed to 0.1 μM, but a gradual decrease with increasing pollutant concentrations (Figures 4A, C). Treatment with 0.1 μM−600 μM PFOS for 48 h caused significantly decreased cell viability (Figure 4B).

As shown in Figures 4A–C, cell viability was ~80% compared to the control group, after incubating the KGN cells with 100 μM PFOS, 400 μM PFOA, and PFHpA for 48 h (*p* < 0.05, Supplementary Table S8). Consequently, these concentrations were used in the following experiments. The effects of PFOA, PFOS, and PFHpA exposure on AMH, E_2_, T, and P levels are shown in Figures 4D–G. Compared with the control group, the concentration of E_2_ remarkably decreased after treatment with 0.1 μM−100 μM PFAS (Figure 4E). There was no statistical difference in the changes of other hormones (Figure 4).

## 4 Discussion

In this case-control study, 96 follicular fluid samples were collected and 24 PFAS were detected. After adjusting for potential confounders, the multivariable logistic regression analysis indicated a significant association between the middle levels of PFHxA in follicular fluid and elevated odds of PCOS. In the cases, a negative relationship between PFHxA and LH concentrations was observed. PFOA was positively associated with E_2._ Among controls, PFOS was positively associated with P, but negatively associated with AMH. The mediation effect of LH in the association of PFHxA exposure and PCOS was not statistically significant. Moreover, in the cell experiments, PFOA, PFOS, and PFHpA exposure decreased the levels of E_2_.

PFAS has been measured in follicular fluid in few studies. PFAS can bind to proteins that are capable of crossing the blood follicle barrier, such as B-lipoproteins, albumin, and liver fatty acid-binding proteins, and therefore easily transported from the bloodstream into the follicle (32, 33). In general, the PCOS cases had higher levels of PFAS compared with the controls (Table 2). These discrepancies between the two groups are likely due to the different menstrual characteristics of the participants. Menstruation could play a crucial role in the elimination of PFAS from the body, and it has been suggested that this process might explain lower PFAS concentrations in women of reproductive age compared to males and in post-menopausal women (34, 35). For PCOS women experiencing oligomenorrhea or amenorrhoea, similar levels of exposure could lead to higher follicular fluid PFAS concentrations. Additionally, in our samples, PFOA and PFOS were the two most abundant PFAS, with their concentrations being higher than those in follicular fluid samples from Peking University People's Hospital (Beijing, China; median: PFOA: 3.38 ng/mL; PFOS: 4.54 ng/mL), but lower than those from the International Peace Maternity and Child Health Hospital (Shanghai, China; median: PFOA: 7.04 ng/mL; PFOS: 5.99 ng/mL) (36, 37). This suggested that geographic differences may influence PFAS exposure. PFAS were widespread in the coastal waters of China, and heavily influenced by the locations of fluorochemical manufacturing facilities (3). Du et al. explored the distribution patterns of 12 PFAS in 91 main river estuaries along the entire coast of China for the first time. The results showed that PFOA, PFBA, and PFBS were dominant in the whole coastal region (3). Another study, conducted near two PFAS manufacturing plants in Fujian, China, found that the predominant compounds among the surface water, sediment, and fish were PFBS, PFOS, and PFOA (38). These results were inconsistent with our findings. In our investigation, the detection rate of PFBA and PFBS was <10%. More research is required to determine the causes of this discrepancy.

A pronounced correlation was observed among PFOA, PFNA, PFDA, PFUnDA, PFOS, and PFHxS, with correlation coefficients ranging from ~0.42 to 0.86 (Supplementary Table S5). These ratios were similar to those documented by Wang et al. (coefficients: 0.32 to 0.89) (17), suggesting that individuals might be exposed to these PFAS in a similar pattern. These substances are categorized as PFAAs, which are frequently present in a variety of everyday products, including nonstick cookware and food packaging (6). According to a previous study, children who regularly consume fish had considerably higher concentrations of PFAAs, such as PFDA, PFUnDA, PFHxS, PFOS, and PFNA, suggesting that fish/shellfish consumption is one source of human exposure to these PFAAs (39). In addition, the carboxylic acid group in PFOA, PFNA, PFDA, and PFUnDA is the same, while the carbon chain length progressively grows. The sulfonic acid group is present in PFOS and PFHxS. The similarities in structures and protein-binding characteristics in serum of these PFAS could lead to comparable distribution and excretion profiles (40, 41).

The restricted production and utilization of PFOA and PFOS have indirectly prompted the development of alternative substances in the manufacturing of PFAS, such as short-chain PFAS and fluorotelomer sulfonates (FTS), which include PFHxA, 4:2 FTS, 6:2 FTS, and 8:2 FTS (42–44). Since the 1970s, these compounds have been widely used in paints, adhesives, polishes, and other industrial products (45, 46). In recent years, such emerging alternatives were reported in groundwater (47), indoor dust (48), and marine organisms (49). Several studies have also observed similar or more toxic potency of these alternatives compared with PFOA and PFOS (50). In the present study, the detection rates of PFHxA, 4:2 FTS, 6:2 FTS, and 8:2 FTS were all above 75%. The association between PFHxA and increased odds of PCOS was found to be statistically significant (*p* = 0.013), consistent with an earlier study by Zhan et al. (8). They also observed the positive associations between PFOS, PFDoA, 6:2 Cl-PFESA, and HFPO-DA in plasma and PCOS prevalence. The association of PFOS exposure with PCOS was not significant in our investigation, probably owing to differences in internal exposure levels, and underlying mechanisms in various matrices. Additionally, a case-control study regarding diminished ovarian reserve (DOR) suggested that PFHxA in follicular fluid might elevate the risk of DOR (51). *In vivo* experiments have also shown that female progeny from mice exposed to PFHxA displayed symptoms of impaired ovarian functions, including reduced ovarian size and a lower count of ovarian follicles (52). These results suggested that PFHxA might increase the risk of PCOS in women of reproductive age. We also found that 6:2 FTS was negatively associated with PCOS. Li et al. conducted a study to compare the toxicity of PFOS and 6:2 FTS using the common invertebrate Eisenia fetida in soil (53). The findings indicated that PFOS and 6:2 FTS caused oxidative stress and apoptosis in earthworms, resulting in developmental and reproductive toxicity. Emerging PFAS concentrations in biomonitoring studies of the susceptible population and the information of their toxicity are limited (54). Therefore, systematic studies were required to validate the potential effects of emerging PFAS exposure on women with PCOS.

Our study observed inconsistent results using the BKMR model in which overall follicular fluid PFAS were inversely associated with the risk of PCOS after adjusting for covariates. This tendency was primarily caused by PFHxA and 6:2 FTS, of which the PIP values were 0.983 and 0.975, respectively. Another mixture analysis found a correlation between higher plasma PFAS and higher risk of PCOS (8). The different PFAS levels and confounding factors could be the reason for the discrepancies in the outcomes. Some characteristics that influence PFAS elimination and may also be associated with PCOS, should be taken into account covariates in the future assessments of associations between PFAS concentrations and reproductive outcomes.

In women diagnosed with PCOS, the gonadotropin profile typically reveals elevated serum levels of LH and the LH to FSH ratio (55). Similar characteristics were also observed in our study, with the median concentration of LH and LH/FSH in the cases being 1–2 times that in the controls (median: LH: 6.34 mIU/mL vs. 3.94 mIU/mL; LH/FSH: 1.06 vs. 0.59). In the assessment of associations between PFAS levels and sex steroid hormones, PFHxA levels in follicular fluid were shown to be adversely associated with LH in the cases. To evaluate the possible mediating role of LH in the association between PFHxA and PCOS, the mediation analysis was conducted. The direct effect of PFHxA was significant (*p* < 0.05), suggesting that PFHxA was more likely to play a direct role in the development of PCOS rather than being mediated by LH. In a group of 251 women with PCOS and 48 healthy control women, a retrospective study on serum levels of gonadotropin levels found that the LH-FSH ratio serves as an important diagnostic instrument in the assessment of women with PCOS (56), suggesting the change in LH levels was not due to any exposure. In addition, a hypothesis-driven weight-of-evidence (WoE) analysis indicated that exposure to PFHxA did not result in negative outcomes associated with changes in endocrine function (52). There was no evidence to suggest that any adverse outcomes observed with exposure to PFHxA are attributable to alterations in LH.

Even though many factors critically affect follicle development, FSH and LH have a central role in regulating the complex endocrine mechanisms of the ovaries. Both hormones are synthesized and released by the same pituitary cells (57). The two cell-two gonadotrophin theory indicates that LH receptors are mainly located at the plasma membrane of the internal theca cells, while FSH receptors are expressed in the granulosa cells. LH stimulates theca cells to secrete androgens, which migrate to granulosa cells through the basal plate of follicles, whereas FSH stimulates P and E_2_ secretion from granulosa cells (58). Studies have reported that E_2_ could improve follicle survival, growth, and oocyte health (59). In PCOS patients, the statistical correlations between PFOS, PFOA, PFHpA, and steroid hormones suggested that they could increase the sensitivity to endocrine effects, with the beta coefficients ranging from 0.50 to 0.76 (Table 4). To further verify the relationship between PFAS and steroid hormones, PFOS, PFOA, PFHpA, and the KGN cell line was used as an *in vitro* model to investigate the effect of PFAS on human ovarian granulosa cells. When the exposure concentration is >0.1 μM, the cell viability gradually decreased with increasing PFOA and PFHpA concentrations. At the same time, PFOS, PFOA, and PFHpA exposure remarkably decreased the concentration of E_2_ (Figure 4), suggesting that PFAS may reduce the quality of oocytes by inhibiting the secretion of E_2_ by ovarian granulosa cells. Based on some findings, the KGN cell line is regarded as a valuable model for understanding the regulation of steroidogenesis in human granulosa cells and follicle development (29). In agreement with our findings, a retrospective study found that the PCOS groups had lower E_2_ levels in follicular fluid (60). An animal experiment found that chronic exposure to PFOS inhibited the biosynthesis of E_2_, thereby adversely affecting follicular development and ovulation (23). The potential mechanism may be that PFAS inhibition enzyme activities of the estrogen biosynthesis (61, 62). Mutalifu et al. explored the potential reproductive toxicity of perfluoro-(3,5,7,9-tetraoxadecanoic) acid (PFO4DA). Mechanistic analysis revealed that PFO4DA dramatically suppressed the expression levels of steroidogenic acute regulatory protein (StAR) and cytochrome P450 family 11 subfamily A member 1 (CYP11A1) in mice (63). In the research of Tatarczuch et al., the PFAS combination decreased the expression of steroidogenic enzyme 3-beta-hydroxysteroid dehydrogenase (3βHSD) and the production of the estradiol from granulosa cells (64). A quantitative structure-activity relationship and molecular docking analysis showed that PFOS, PFOA, and PFHpA at a concentration of 100 μM substantially inhibited human 17β-Hydroxysteroid dehydrogenases (17β-HSD1) activity (65). There are currently fewer molecular studies of the PFAS endocrine pathway. Further research is required to confirm the effect of PFAS on E_2_ and to assess the biological basis for the effect.

## 5 Limitations

This study conducts an assessment of follicular fluid PFAS concentrations in a potentially sensitive population, PCOS patients, in relationship to PCOS and a range of steroid hormones. However, a few limitations existed in this study. First, acquiring suitable follicular fluid samples was challenging. Our sample size may not be large enough to provide adequate statistical power for assessing the variations in some of the PFAS concentrations between patients with PCOS and controls. Secondly, menstrual characteristics, reproductive history, and daily habits can also alter the concentrations of PFAS in follicular fluid (12, 66). The potential confounding effects of by unmeasured variables still may have introduced bias into our findings. Thus, the results presented can be considered exploratory. It is imperative to take into account more confounding factors and further investigate the underlying mechanisms of PFAS on reproductive health in the future.

## 6 Conclusion

In our study, 24 PFAS were detected in the follicular fluid of PCOS cases who had undergone fertility treatment. PFHxA in follicular fluids is associated with an increased risk of PCOS. Combined with *in vitro* experiments, steroid hormones might play a role in the pathogenesis of PCOS after exposure to environmentally relevant PFAS. These findings have enhanced our comprehension of the harmful effects of PFHxA exposure and have sparked worries regarding the impact of exposure to emerging PFAS on reproductive health.

## Data Availability

The original contributions presented in the study are included in the article/Supplementary material, further inquiries can be directed to the corresponding author.
