# Supplementary material for: Associations of per- and polyfluoroalkyl substances in follicular fluid with polycystic ovarian syndrome in infertile women may be mediated by sex hormones
Source: Front Public Health. 2025 Jun 10;13:1526918. doi: 10.3389/fpubh.2025.1526918 (PMC12185472; doi:10.3389/fpubh.2025.1526918)
Supplement: Supplementary file 1 [file Data_Sheet_1.docx]

Supplementary information and Table Captions

**1.Analysis for PFAS**

**2.Quality Control**

**Supplementary Table 1.** Mobile phase gradient elution procedure.

**Supplementary Table 2.** Twenty-four PFAS chromatographic retention time, transitions and MS/MS conditions.

**Supplementary Table 3.** Linearity ranges, r^2^, LODs and LOQs of the PFAS.

**Supplementary Table 4.** Accuracy and precision of the spiked recoveries of 24 PFAS measured at three different concentration levels (n=6).

**Supplementary Table 5.** Spearman correlation coefficients between PFAS concentrations in follicular fluid (n=96).

**Supplementary Table 6.** Spearman correlation coefficients between PFAS concentration in follicular fluid and clinical characteristics (n=96).

**Supplementary Table 7.** Posterior inclusion probabilities (PIPs) of each PFAS in BKMR.

**Supplementary Table 8.** Compared with the control, P values for the cell viability under different concentrations of PFAS. The KGN cells were incubated for 48 h.

**1.Analysis for PFAS**

In brief, 500 μL follicular fluid was transferred to a 15 mL polypropylene (PP) tube (Thermo Scientific, USA), followed by the addition of 500 μL water and 20 μL internal standards (250 μg/L, ^13^C_8_-PFOA, and ^13^C_4_-MPFOS). The samples were extracted with methyl-tert-butyl ether (MTBE), and then centrifuged. The collected supernatants were allowed to evaporate to dryness under nitrogen at 40 ℃ and reconstituted in 0.5 mL methanol/water (50:50, v/v). Finally, the reconstituted samples were centrifuged at 17450 rpm for 15 min, and the supernatants were removed for liquid chromatography-tandem mass spectrometry (LC-MS/MS) (8050, Shimadzu, Japan) analysis with electrospray ionization (ESI) interface operating in negative mode. Chromatographic separation of the analytes was achieved with an AccucoreTM C_18_ column (100 mm × 4.6 mm internal diameter, 2.6 μm particle size, Thermo Fisher Scientific, USA) and an Accucore C_18_ guard column (10 mm × 4.6 mm internal diameter, Thermo Fisher Scientific), maintained at 35 ℃, with a flow rate of 0.40 mL/min and injection volume of 5 μL. Mobile phases consisted of 2 mM ammonium acetate in water (A) and methanol (B). Calibration curves ranged from 0 to 100 μg/L and exhibited excellent linearity, with R^2^ > 0.99. Further details of mobile phase and mass spectrometry settings are provided in Tables S1 and S2.

**2. Quality Control**

To avoid contamination, all accessible polytetrafluoroethylene materials were prohibited in the present study. Procedural blanks (n = 6) and method blanks (n = 6) spiked with labeled internal standards were analyzed for each batch of samples. The target compounds were quantified using the isotope dilution method with methanol/water (50:50, v/v) at concentrations of 0, 0.2, 1, 5, 10, 25, 50, and 100 μg/L. Low-concentration calibration curves were used to determine the limits of detection (LOD) and quantification (LOQ). LOD and LOQ were defined as a signal-to-noise ratio of 3 and 10, respectively. The matrix-spiked recoveries (n = 6) were obtained by spiking at three concentrations (5, 10, and 50 μg/L). The details of LOD and LOQ, relative standard deviation, and matrix-spiked recoveries are listed in Tables S3 and S4. PFAS concentrations below the LOD were estimated as the LOD divided by the square root of 2.

**Supplementary Table 1.** Mobile phase gradient elution procedure.

| **Time (min)** | **Flow rate (mL/min)** | **A (%)** | **B (%)** |
| --- | --- | --- | --- |
| 0.01 | 0.40 | 80 | 20 |
| 5.00 | 0.40 | 0 | 100 |
| 9.00 | 0.40 | 0 | 100 |
| 9.50 | 0.40 | 80 | 20 |
| 12.0 | 0.40 | 80 | 20 |

Note: Solvent A: 2 mM ammonium acetate in water; Solvent B: methanol.

**Supplementary Table 2.** 24 PFAS chromatographic retention time, transitions and MS/MS conditions.

| **PFAS** | **Molecular mass** | **Parent Ion**  **(m/z)** | **Product Ion**  **(m/z)** | **Retention time**  **(min)** | **Collision Energy** | **Dwell time**  **(ms)** |
| --- | --- | --- | --- | --- | --- | --- |
| **^*^PFOA** | 414.07 | 413.05 | 369.00 | 7.10 | 11.0 | 20 |
| **PFOA** | 414.07 | 413.05 | 169.05 | 7.10 | 19.0 | 20 |
| **PFBA** | 214.04 | 213.05 | 169.00 | 5.55 | 12.0 | 20 |
| **PFPeA** | 264.65 | 263.05 | 218.95 | 6.20 | 9.0 | 20 |
| **PFHxA** | 314.05 | 313.05 | 269.05 | 6.59 | 10.0 | 20 |
| **^*^PFHpA** | 364.06 | 363.05 | 319.10 | 6.60 | 11.0 | 20 |
| **PFHpA** | 364.06 | 363.05 | 119.20 | 6.49 | 22.0 | 20 |
| **^*^PFNA** | 464.08 | 463.05 | 419.00 | 7.29 | 12.0 | 20 |
| **PFNA** | 464.08 | 463.05 | 218.90 | 7.29 | 19.0 | 20 |
| **^*^PFDA** | 514.08 | 513.05 | 468.95 | 7.47 | 13.0 | 20 |
| **PFDA** | 514.08 | 513.05 | 218.95 | 7.44 | 18.0 | 20 |
| **^*^PFUnDA** | 564.09 | 563.10 | 519.00 | 7.60 | 13.0 | 20 |
| **PFUnDA** | 564.09 | 563.10 | 269.10 | 7.60 | 20.0 | 20 |
| **^*^PFDoDA** | 614.10 | 613.10 | 568.95 | 7.50 | 14.0 | 20 |
| **PFDoDA** | 614.10 | 613.10 | 169.20 | 7.39 | 29.0 | 20 |
| **^*^PFTrDA** | 664.10 | 663.10 | 618.90 | 7.84 | 13.0 | 20 |
| **PFTrDA** | 664.10 | 663.10 | 269.20 | 7.84 | 20.0 | 20 |
| **^*^PFTeDA** | 714.11 | 713.10 | 668.85 | 7.95 | 15.0 | 20 |
| **PFTeDA** | 714.11 | 713.10 | 218.90 | 7.95 | 25.0 | 20 |
| **^*^PFOS** | 500.00 | 499.00 | 80.00 | 7.27 | 49.0 | 20 |
| **PFOS** | 500.00 | 499.00 | 99.05 | 7.27 | 41.0 | 20 |
| **PFBS** | 300.00 | 299.00 | 80.00 | 6.27 | 35.0 | 20 |
| **^*^PFPeS** | 350.00 | 349.00 | 80.00 | 6.61 | 45.0 | 20 |
| **PFPeS** | 350.00 | 349.00 | 99.00 | 6.61 | 31.0 | 20 |
| **^*^PFHxS** | 400.00 | 399.00 | 79.95 | 6.87 | 46.0 | 20 |
| **PFHxS** | 400.00 | 399.00 | 99.00 | 6.87 | 35.0 | 20 |
| **^*^PFHpS** | 450.00 | 449.00 | 80.00 | 7.10 | 49.0 | 20 |
| **PFHpS** | 450.00 | 449.00 | 99.00 | 7.09 | 39.0 | 20 |
| **^*^4:2 FTS**  **4:2 FTS** | 350.14  328.00 | 349.15  327.00 | 80.00  307.00 | 6.60  6.55 | 45.0  19.0 | 20  20 |
| **^*^6:2 FTS**  **6:2 FTS** | 450.15  450.15 | 449.15  449.15 | 80.00  99.00 | 7.09  7.09 | 45.0  38.0 | 20  20 |
| **^*^8:2 FTS**  **8:2 FTS** | 550.17  528.00 | 549.15  527.00 | 80.15  506.95 | 7.50  7.47 | 50.0  27.0 | 20  20 |
| **^*^PFOSA** | 499.15 | 498.15 | 78.00 | 8.27 | 45.0 | 20 |
| **PFOSA** | 499.15 | 498.15 | 169.05 | 8.27 | 30.0 | 20 |
| **^*^PFNS** | 550.00 | 549.00 | 79.90 | 7.45 | 55.0 | 20 |
| **PFNS** | 550.00 | 549.00 | 98.85 | 7.45 | 46.0 | 20 |
| **^*^PFDS** | 600.00 | 599.00 | 79.90 | 7.58 | 55.0 | 20 |
| **PFDS** | 600.00 | 599.00 | 99.10 | 7.58 | 52.0 | 20 |
| **^*^N_MeFOSAA**  **N_MeFOSAA** | 571.00  571.00 | 570.00  570.00 | 418.85  219.00 | 7.54  7.54 | 22.0  27.0 | 20  20 |
| ***N_EtFOSAA** | 585.24 | 584.25 | 419.00 | 7.61 | 22.0 | 20 |
| ***N_EtFOSAA** | 585.24 | 584.25 | 218.90 | 7.61 | 20.0 | 20 |
| **^*13^C-PFOA** | 422.00 | 421.00 | 375.99 | 7.10 | 10.0 | 20 |
| **^13^C-PFOA** | 422.00 | 421.00 | 172.20 | 7.10 | 20.0 | 20 |
| **^*13^C-PFOS** | 504.00 | 503.00 | 79.95 | 7.28 | 54.0 | 20 |
| **^13^C-PFOS** | 504.00 | 503.00 | 99.00 | 7.28 | 45.0 | 20 |

Note: For most analytes, the first one marked as “^*^” was used for quantitation (quantifier) and the second one was used for confirmation (qualifier).

**Supplementary Table 3.** Linearity ranges, r^2^, LODs and LOQs of the PFAS.

| **PFAS** | **Linearity range (μg/L)** | **Correlation coefficent (r^2^)** | **LOD (μg/L)** | **LOQ (μg/L)** |
| --- | --- | --- | --- | --- |
| **PFOA** | 0-100 | 0.994 | 0.053 | 0.167 |
| **PFBA** | 0-100 | 0.993 | 0.087 | 0.263 |
| **PFPeA** | 0-100 | 0.997 | 0.055 | 0.192 |
| **PFHxA** | 0-100 | 0.996 | 0.068 | 0.202 |
| **PFHpA** | 0-100 | 0.992 | 0.060 | 0.177 |
| **PFNA** | 0-100 | 0.996 | 0.057 | 0.168 |
| **PFDA** | 0-100 | 0.995 | 0.100 | 0.295 |
| **PFUnDA** | 0-100 | 0.992 | 0.080 | 0.240 |
| **PFDoDA** | 0-100 | 0.996 | 0.055 | 0.168 |
| **PFTrDA** | 0-100 | 0.991 | 0.066 | 0.200 |
| **PFTeDA** | 0-100 | 0.983 | 0.150 | 0.355 |
| **PFOS** | 0-100 | 0.992 | 0.217 | 0.657 |
| **PFBS** | 0-100 | 0.993 | 0.077 | 0.232 |
| **PFPeS** | 0-100 | 0.994 | 0.140 | 0.420 |
| **PFHxS** | 0-100 | 0.994 | 0.132 | 0.398 |
| **PFHpS** | 0-100 | 0.995 | 0.148 | 0.447 |
| **4:2 FTS** | 0-100 | 0.993 | 0.154 | 0.470 |
| **6:2 FTS** | 0-100 | 0.992 | 0.220 | 0.660 |
| **8:2 FTS** | 0-100 | 0.991 | 0.068 | 0.203 |
| **PFOSA** | 0-100 | 0.998 | 0.013 | 0.030 |
| **PFNS** | 0-100 | 0.997 | 0.248 | 0.747 |
| **PFDS** | 0-100 | 0.989 | 0.205 | 0.620 |
| **N_MeFOSAA** | 0-100 | 0.997 | 0.030 | 0.095 |
| **N_EtFOSAA** | 0-100 | 0.999 | 0.035 | 0.105 |

Note: LOD: limits of detection; LOQ: limits of quantification.

**Supplementary Table 4.** Accuracy and precision of the spiked recoveries of 24 PFAS measured at three different concentration levels (n=6).

| **PFAS** | **Spiked levels** | | | | | | | | |
| --- | --- | --- | --- | --- | --- | --- | --- | --- | --- |
|  | **5 (μg/L)** | |  | **10 (μg/L)** | |  | **50 (μg/L)** | |  |
|  | **Recovery (%)** | **RSD (%)** |  | **Recovery (%)** | **RSD (%)** |  | **Recovery (%)** | **RSD (%)** |  |
| **PFOA** | 97.0 | 7.31 |  | 107 | 13.3 |  | 111 | 7.86 |  |
| **PFBA** | 78.9 | 0.74 |  | 75.7 | 1.74 |  | 80.5 | 1.52 |  |
| **PFPeA** | 89.0 | 4.47 |  | 85.0 | 5.74 |  | 85.0 | 2.60 |  |
| **PFHxA** | 75.9 | 6.75 |  | 84.1 | 1.82 |  | 90.3 | 1.62 |  |
| **PFHpA** | 86.7 | 3.29 |  | 92.6 | 2.85 |  | 94.4 | 2.01 |  |
| **PFNA** | 111 | 4.34 |  | 112 | 1.80 |  | 119 | 1.39 |  |
| **PFDA** | 83.1 | 4.67 |  | 74.9 | 2.58 |  | 83.8 | 3.23 |  |
| **PFUnDA** | 129 | 7.07 |  | 116 | 4.14 |  | 126 | 1.88 |  |
| **PFDoDA** | 105 | 10.2 |  | 98.6 | 7.04 |  | 96.3 | 5.09 |  |
| **PFTrDA** | 74.3 | 9.86 |  | 72.6 | 3.31 |  | 77.6 | 6.23 |  |
| **PFTeDA** | 80.2 | 5.17 |  | 61.7 | 4.73 |  | 50.2 | 0.75 |  |
| **PFOS** | 91.2 | 3.12 |  | 97.8 | 7.14 |  | 108 | 2.17 |  |
| **PFBS** | 100 | 3.76 |  | 102 | 3.23 |  | 95.4 | 3.30 |  |
| **PFPeS** | 78.1 | 4.47 |  | 84.8 | 6.00 |  | 85.2 | 1.51 |  |
| **PFHxS** | 80.3 | 6.96 |  | 85.7 | 3.27 |  | 88.3 | 2.18 |  |
| **PFHpS** | 99.2 | 7.18 |  | 102 | 6.78 |  | 102 | 2.86 |  |
| **4:2 FTS** | 95.3 | 1.76 |  | 92.5 | 3.64 |  | 93.5 | 3.19 |  |
| **6:2 FTS** | 102 | 5.33 |  | 102 | 5.88 |  | 106 | 2.10 |  |
| **8:2 FTS** | 95.3 | 8.06 |  | 110 | 8.31 |  | 114 | 9.51 |  |
| **PFOSA** | 65.9 | 1.43 |  | 73.9 | 5.67 |  | 83.7 | 5.26 |  |
| **PFNS** | 115 | 11.2 |  | 108 | 2.71 |  | 118 | 7.72 |  |
| **PFDS** | 99.0 | 7.31 |  | 91.3 | 6.18 |  | 97.1 | 9.21 |  |
| **N_MeFOSAA** | 70.1 | 4.50 |  | 67.5 | 3.07 |  | 74.0 | 2.91 |  |
| **N_EtFOSAA** | 88.6 | 8.41 |  | 83.3 | 1.22 |  | 86.7 | 1.40 |  |

Note: RSD: relative standard deviation.

**Supplementary Table 5.** Spearman correlation coefficients between PFAS concentrations in follicular fluid (n=96).

| **PFAS** | **PFOA** | **PFPeA** | **PFHxA** | **PFHpA** | **PFNA** | **PFDA** | **PFUnDA** | **PFTrDA** | **PFOS** | **PFPeS** | **PFHxS** | **4:2 FTS** | **6:2 FTS** | **8:2 FTS** |
| --- | --- | --- | --- | --- | --- | --- | --- | --- | --- | --- | --- | --- | --- | --- |
| **PFOA** | 1.000 |  |  |  |  |  |  |  |  |  |  |  |  |  |
| **PFPeA** | -0.037 | 1.000 |  |  |  |  |  |  |  |  |  |  |  |  |
| **PFHxA** | -0.037 | **0.273^**^** | 1.000 |  |  |  |  |  |  |  |  |  |  |  |
| **PFHpA** | 0.186 | -0.116 | -0.126 | 1.000 |  |  |  |  |  |  |  |  |  |  |
| **PFNA** | **0.633^**^** | -0.103 | 0.002 | -0.108 | 1.000 |  |  |  |  |  |  |  |  |  |
| **PFDA** | **0.497^**^** | -0.140 | -0.060 | -0.110 | **0.864^**^** | 1.000 |  |  |  |  |  |  |  |  |
| **PFUnDA** | **0.447^**^** | -0.055 | -0.047 | -0.149 | **0.829^**^** | **0.864^**^** | 1.000 |  |  |  |  |  |  |  |
| **PFTrDA** | **0.408^**^** | **-0.203^*^** | -0.042 | -0.085 | **0.638^**^** | **0.651^**^** | **0.682^**^** | 1.000 |  |  |  |  |  |  |
| **PFOS** | **0.420^**^** | -0.174 | -0.146 | 0.016 | **0.678^**^** | **0.722^**^** | **0.712^**^** | **0.519^**^** | 1.000 |  |  |  |  |  |
| **PFPeS** | -0.030 | -0.038 | -0.097 | 0.181 | **-0.222^*^** | **-0.264^**^** | **-0.220^**^** | -0.123 | **-0.246^*^** | 1.000 |  |  |  |  |
| **PFHxS** | **0.717^**^** | -0.061 | -0.131 | 0.076 | **0.612^**^** | **0.536^**^** | **0.531^**^** | **0.496^**^** | **0.536^**^** | 0.035 | 1.000 |  |  |  |
| **4:2 FTS** | -0.109 | -0.043 | 0.055 | 0.131 | **-0.255^*^** | **-0.347^**^** | **-0.317^**^** | **-0.254^*^** | **-0.243^*^** | **0.646^**^** | -0.100 | 1.000 |  |  |
| **6:2 FTS** | **0.211^*^** | -0.178 | -0.065 | -0.018 | **0.346^**^** | **0.334^**^** | **0.334^**^** | **0.296^**^** | **0.468^**^** | -0.141 | **0.291^*^** | -0.102 | 1.000 |  |
| **8:2 FTS** | 0.129 | 0.157 | 0.027 | 0.063 | 0.173 | 0.168 | 0.114 | -0.011 | **0.219^*^** | 0.036 | **0.221^*^** | 0.001 | 0.000 | 1.000 |

Note: ^*^*p* < 0.05; ^**^*p* < 0.01; ^***^*p* < 0.001.

**Supplementary Table 6.** Spearman correlation coefficients between PFAS concentration in follicular fluid and clinical characteristics.(n=96).

|  | **AMH** | **FSH** | **LH** | **LH/FSH** | **E_2_** | **T** | **P** | **PRL** | **FBG** |
| --- | --- | --- | --- | --- | --- | --- | --- | --- | --- |
| **PFOA** | -0.084 | 0.078 | -0.117 | 0.130 | -0.018 | -0.123 | -0.004 | 0.030 | 0.026 |
| **PFPeA** | -0.131 | 0.101 | -0.134 | **-0.228^*^** | 0.155 | -0.045 | 0.038 | -0.118 | -0.101 |
| **PFHxA** | -0.107 | -0.128 | -0.193 | -0.087 | 0.075 | -0.162 | -0.144 | 0.136 | **-0.224^*^** |
| **PFHpA** | -0..052 | 0.138 | 0.119 | 0.018 | -0.120 | -0.108 | 0.072 | -0.107 | -0.002 |
| **PFNA** | 0.024 | 0.013 | 0.029 | 0.195 | -0.119 | -0.107 | 0.022 | 0.014 | -0.009 |
| **PFDA** | 0.005 | 0.007 | 0.088 | 0.199 | -0.041 | -0.058 | 0.042 | 0.082 | 0.000 |
| **PFUnDA** | 0.016 | -0.013 | 0.028 | 0.175 | -0.047 | -0.140 | -0.001 | 0.039 | 0.018 |
| **PFTrDA** | -0.007 | 0.039 | 0.066 | **0.207^*^** | -0.061 | -0.162 | -0.102 | 0.166 | -0.024 |
| **PFOS** | -0.058 | -0.058 | 0.103 | 0.191 | -0.147 | -0.109 | 0.017 | -0.099 | 0.073 |
| **PFPeS** | -0.018 | 0.062 | -0.139 | -0.060 | 0.096 | 0.142 | 0.066 | 0.032 | -0.103 |
| **PFHxS** | -0.105 | 0.148 | -0.047 | 0.060 | -0.066 | -0.170 | -0.032 | 0.074 | -0.014 |
| **4:2 FTS** | 0.038 | -0.078 | -0.032 | 0.078 | -0.008 | 0.123 | -0.059 | -0.035 | -0.138 |
| **6:2 FTS** | 0.000 | **-0.202^*^** | 0.044 | **0.226^*^** | -0.196 | 0.025 | 0.005 | 0.102 | -0.022 |
| **8:2 FTS** | -0.140 | **0.215^*^** | -0.025 | 0.012 | -0.121 | -0.004 | 0.051 | **-0.222^*^** | 0.018 |

**Note**: *p < 0.05, **p < 0.01.

**Supplementary Table 7.** Posterior inclusion probabilities (PIPs) of each PFAS in BKMR.

| **PFAS** | **PFOA** | **PFPeA** | **PFHxA** | **PFHpA** | **PFNA** | **PFDA** | **PFUnDA** | **PFTrDA** | **PFOS** | **PFPeS** | **PFHxS** | **4:2**  **FTS** | **6:2**  **FTS** | **8:2**  **FTS** |
| --- | --- | --- | --- | --- | --- | --- | --- | --- | --- | --- | --- | --- | --- | --- |
| **PIPs** | 0.372 | 0.973 | 0.983 | 0.669 | 0.632 | 0.523 | 0.481 | 0.443 | 0.452 | 0.579 | 0.371 | 0.657 | 0.975 | 0.472 |

**Note:** All PFAS were ln-transformed. Models were adjusted for age, BMI and employment status.

**Supplementary Table 8.** Compared with the control, P values for the cell viability under different concentrations of PFOA, PFOS and PFHpA. The KGN cells were incubated for 48 h.

| **PFAS**  **(μM)** | **0.1** | **1** | **10** | **100** | **200** | **400** | **600** |
| --- | --- | --- | --- | --- | --- | --- | --- |
| **PFOA** | < 0.0001 | 0.9878 | > 0.9999 | 0.9811 | 0.8211 | 0.0036 | < 0.0001 |
| **PFOS** | < 0.0001 | < 0.0001 | < 0.0001 | < 0.0001 | < 0.0001 | < 0.0001 | < 0.0001 |
| **PFHpA** | 0.1957 | 0.2341 | 0.9924 | 0.9990 | 0.8632 | 0.0156 | < 0.0001 |

**Note:** Statistical significance was set at *p* < 0 05.
